# Complete chloroplast genome sequence of the medicinal plant *Gynostemma pentaphyllum*

**DOI:** 10.1080/23802359.2016.1197054

**Published:** 2016-10-29

**Authors:** Xin-Yi Zeng, Li-Qiu Zou, Xue-Jun Kuang, Ying Li

**Affiliations:** Institute of Medicinal Plant Development, China Academy of Medical Sciences & Peking Union Medical College, Beijing, China

**Keywords:** Chloroplast genome, *Gynostemma pentaphyllum*, phylogenetic relationship

## Abstract

We report the complete chloroplast genome sequence of *Gynostemma pentaphyllum*, a well-known traditional Chinese medicine, which produces triterpenoid saponins similar to Panax ginseng. The assembled chloroplast genome (cpDNA) was 157,654 bp in length and structurally divided into four distinct regions, namely, large single copy region (86,794 bp), small single copy region (18,654 bp) and a pair of inverted repeat regions (26,103 bp). A total of 143 genes were annotated, including 87 protein-coding genes, 10 tRNA genes and 46 rRNA genes. Phylogenetic analysis revealed that the chloroplast genome sequence of *G. pentaphyllum* is most closely related to Cucumis melo.

*Gynostemma pentaphyllum* is a perennial creeping herb of the genus *Gynostemma* in the Cucurbitaceae family and is widely distributed throughout China, India, Myanmar, Korea and Japan (Chen 1995). *G. pentaphyllum* is a traditional Chinese medicinal herb that contains more than 100 dammarane-type saponins (Yin et al. 2004; Liu et al. 2005). These saponins possess important biological functions and are responsible for its beneficial effects against inflammation, hyperlipidemia, cardiovascular disease and cancer (Attawish et al. 2004; Circosta et al. 2005; Wang et al. 2007; Yan et al. 2014). Elucidation of the complete *G. pentaphyllum* cp genome would enrich the chloroplast genome information of the Cucurbitaceae family and contribute to further research regarding its evolution and molecular identification.

The total DNA was isolated from the mature leaves of *G. pentaphyllum*, which were collected from the base of Good Agricultural Practice of Medicinal Plants and Animals (GAP) for *G. pentaphyllum* in Pingli County, Shanxi Province. Pingli County is one of the major *G. pentaphyllum* producing areas in China. The specimen has been stored in the Institute of Medicinal Plant Development (IMPLAD) and the accession number is Gp_PL01. A Illumina Hiseq 4000 paired-end (PE) genomics library with 150 bp read length was constructed and sequenced. The cp reads from the total data were found by aligning the PE reads to the reference cp genome, *Cucumis sativus* (AJ970307) and *Cucumis melo* (JF412791), which belong to the genus *Cucumis* in the Cucurbitaceae family. Read number analysis indicated that the total DNA contained ∼5% cp DNA. The cp reads were used in the assembly, and the resulting cp genome contigs were manually checked to confirm the ligation between the LSR–IR and SSR–IR boundary. Dual Organellar GenoMe Annotator (DOGMA) (Wyman et al. 2004) was used to predict protein-coding genes, transfer RNA (tRNA) genes and ribosome RNA (rRNA) genes. BLASTX was conducted to identify further the positions of genes with intron by searching against the reference cp genome. The complete cp genome sequence, together with gene annotations, was submitted to the GenBank with accession number KX014626.

The complete chloroplast genome of *G. pentaphyllum* was a circular form of 157,654 bp in length, which was divided into four distinct regions, namely, 86,794 bp of large single copy region, 18,654 bp small single copy region and 26,103 bp of a pair of inverted repeat regions. The chloroplast genome contained annotated genes, including 87 protein-coding genes, 10 tRNA genes and 46 rRNA genes.

A neighbor-joining tree was constructed in MEGA 6.0 (Tamura et al. 2013) using the total chloroplast protein-coding sequences of *G. pentaphyllum*, other Cucurbitales plants and other species. The phylogenetic tree showed that the Cucurbitales, which includes *G. pentaphyllum* and the genus *Cucumis*, was clustered to a branch (Figure 1).

**Figure 1.**
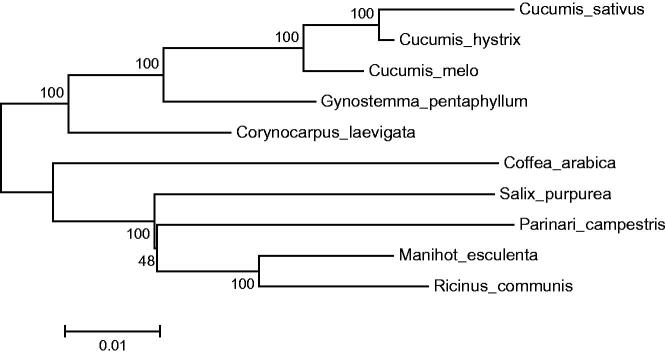
Neighbor-joining tree of the Cucurbitales and related families based on total protein-coding genes. Numbers in the nodes are boot-strap values from 1000 replicates. The chloroplast sequence of *Coffea arabica*, a species of the Gentianales, was set as an outgroup. The GenBank numbers area are as follows: *Cucumis sativus*, AJ970307; *Cucumis hystrix*, KF957866; *Cucumis melo*, JF412791; *Gynostemma pentaphyllum*, KX014626; *Corynocarpus laevigata*, HQ207704; *Coffea arabica*, EF044213; *Salix purpurea*, NC_026722; *Parinari campestris*, NC_024067; *Manihot esculenta*, NC_010433; *Ricinus communis*, JF937588.
